# Explanatory Pluralism and the (Dis)Unity of Science: The Argument from Incompatible Counterfactual Consequences

**DOI:** 10.3389/fpsyt.2016.00032

**Published:** 2016-03-11

**Authors:** Victor Gijsbers

**Affiliations:** ^1^Institute for Philosophy, Universiteit Leiden, Leiden, Netherlands

**Keywords:** explanatory pluralism, unity of science, disunity of science, explanation, counterfactual incompatibility, counterfactuals

## Abstract

What is the relationship between different sciences or research approaches that deal with the same phenomena, for instance, with the phenomena of the human mind? Answers to this question range from a monist perspective according to which one of these approaches is privileged over the others, through an integrationist perspective according to which they must strive to form a unity greater than the sum of its parts, to an isolationist perspective according to which each of them has its own autonomous sphere of validity. In order to assess these perspectives in this article, I discuss the debates about the unity of science and about explanatory pluralism. The most pressing issue turns out to be the choice between the integrative and the isolationist perspective: the question is whether the integrative tendencies in science should be fully indulged in or whether they should be held in check by acknowledging that a certain amount of isolation is necessary. I argue that the issue can be further distilled into the question of whether two true explanations of the same fact can ever fail to be combinable into one single explanation. I show that this can indeed be the case, namely, when the explanations have incompatible counterfactual consequences, something that is often the case when we try to combine explanations from different sciences or research approaches. These approaches thus embody perspectives on the world that are to a certain extent autonomous. This leads to the conclusion that although interdisciplinarity may have many advantages, we should not take the project of integration too far. At the end of the day, the different research approaches with their different perspectives and insights must remain precisely that: different and somewhat disunified.

## Introduction

What is the relationship between the different sciences or, to use a more fine-grained term, research approaches that deal with the human mind? Faced with a variety of explanations for a psychiatric illness – for example, genetic, neurological, cognitive, psychoanalytic, and sociological ­explanations – a scientist could take one of three broad views. First, the view that one explanation will trump all the others, making it the only one that is needed. Second, the view that these explanations can be combined into one unified explanation that is superior to each of the individual ones. Third, the view that these explanations all add to our understanding, but cannot be combined into a single integrated account. In other words, a scientist could take a *monist*, an *integrationist*, or an *isolationist* view. Of course, combinations are also possible: one might believe that genetic and neurological explanations can be integrated, that sociological explanations are useful but will remain isolated, that explanations from cognitive psychology will be trumped by neurological ones, and that psychoanalytic explanations are simply wrong; or any other combination of options.

The distinction between these views has important practical consequences both for research and for therapy. If a monist perspective were correct, scientists and practitioners alike should learn to focus on the specific approach that provides the best explanations (which monists of today are likely to identify with neuroscience and low-level biological approaches to the human brain). But if an integrationist perspective were correct, researchers should focus on doing interdisciplinary research, and practitioners should make sure that they learn to see the interconnections between different clinical approaches and find ways to combine them. Then again, if an isolationist perspective were correct, the most fruitful approach would be one of disciplinary specialization and parallel but isolated lines of treatment.

The general heading under which these issues used to be discussed was that of the *unity of science*, but as I will make clear in Section “From Unity of Science to Explanatory Pluralism,” ­philosophers now generally talk about the issue of *explanatory pluralism*. Those who defend explanatory ­pluralism – which includes almost everyone engaged in the current ­discussion – ­generally reject the monist and isolationist positions in favor of some kind of integrationism, some version of the idea that the different sciences have to work together in order to achieve results that they could not achieve separately. The arguments for this claim come in two flavors: first, arguments to the effect that interdisciplinary research is methodologically superior to monodisciplinary research, and, second, case studies that prove that scientists are actually pursuing such research and achieving their aims through it.

These methodological and empirical approaches are of course very valuable. But in the current paper, I would like to ask a more fundamental question about scientific explanation, namely, whether there is anything in the structure of explanations itself that can form a barrier to their complete integration. If two different research approaches come up with explanations of the same phenomenon, and if these are both true and not logically contradictory, is it then always possible to put them together into a single integrated perspective on the phenomenon? Or is it sometimes the case that we have no choice but to be isolationists, because the explanations themselves just do not fit together? Can true explanations be incompatible? These questions are pertinent. There may be, in our current scientific practice, a presumption in favor of interdisciplinarity and the integration of explanations; but it is far from clear that complete integration is the correct ideal to pursue. Perhaps there is a sense in which, say, a neurological and a sociological explanation of a patient’s symptoms are just “too different” to be forged into a single, more complete explanation.

My task in Section “Combining Explanations” will be to ­analyze the conditions under which two explanations of the same phenomenon can fail to be combinable into a single explanation; in other words, I want to find out what it would mean for two explanations to be “too different.” To this end, I will develop the notion of *counterfactual incompatibility*: the idea that two statements, even though they are logically consistent, can nevertheless imply different things about what would have happened under hypothetical circumstances. I then argue that explanations that are counterfactually incompatible cannot be combined into a single explanation – and far from that being a merely academic possibility, this does in fact regularly happen when we take explanations from different research approaches.

We thus find, from studying the structure of explanations themselves, that there is something in the sciences that resist integration; that, however, much we love interdisciplinarity, there is an extent to which we must remain isolationists; that different research approaches yield perspectives on the world which cannot always be fully integrated. Interdisciplinarity and the search for connections, while commendable, should be held in check by a healthy appreciation of the autonomy of each of the individual approaches scientists are using.

## From Unity of Science to Explanatory Pluralism

The 1930s saw a rising interest in the idea of the unity of science, which is perhaps nowhere more visible than in the activities of one of the fathers of logical empiricism, Otto Neurath. Neurath founded the Unity of Science Institute in 1936; organized a series of conferences between 1935 and 1941 called the International Congresses for the Unity of Science; and started the International Encyclopedia of Unified Science [see Cat (1) for historical details]. Many of the most important philosophers of the era were implicated in one or more of these enterprises, among them Philipp Frank, Charles Morris, Rudolf Carnap, Bertrand Russell, Ernest Nagel, and John Dewey.

Neurath’s own overarching concern with the unity of science movement was to create an environment within which the different sciences could interact and learn from each other. As Pombo et al. (2) put it:

Neurath’s own encyclopedic conception of the unity of science is built on the notion of cooperative action in the scientific community and the accumulation of available results. […] at the heart of the project is the goal of providing a universal medium for communicating across disciplines and languages (p. 4)

But although Neurath’s own view was characterized by symmetric and non-reductionist ideas about communication, cooperation, interdisciplinarity, and interaction between different disciplines (idem, p. 6), the idea of the unity of science was soon interpreted in a much more reductive way. Thus, Oppenheim & Putnam (3) suggests that the unity of science would be achieved when all the terms and all the laws of all the sciences have been reduced to the terms and laws of a single scientific discipline. For Oppenheim and Putnam, the unity of science in this sense is an “over-arching metascientific hypothesis” (p. 6) which, even if it cannot be conclusively shown to be true, is nevertheless credible (p. 8).

It is against this background that we have to understand the position taken up in Fodor’s classic anti-reductionist paper “Special Sciences, or: the Disunity of Science as a Working Hypothesis” (4), namely, the position that it is in all probability useless to search for lawful coextension of predicates from sciences at different levels. If unity of science is understood in the reductionist way that Oppenheim and Putnam understand it, then one is indeed tempted to emphasize, with Fodor, the *disunity* of science rather than its unity. Few scientists are interested in strong reductionist projects, and thus it might seem that they have no reason to seek for a unity of science.

In the philosophy of science, this attitude has been forcefully defended by Dupré (5), Cartwright (6), and Teller (7). They use metaphysical, epistemological, and methodological arguments to argue for a “disunified” and “dappled” view of science in which no overarching, all-encompassing laws can be found, and no single discipline will emerge as foundational. Dupré, in fact, insists that science is not even unified by any common sociological, methodological, or processual element.

We have no quarrel with this general view, but something seems to be missing from it. We are still, and perhaps more than ever, interested in communication, cooperation, interdisciplinarity, and interaction between the sciences; and we share with Oppenheim and Putnam, if not their views about reduction, at least their general aim of “counterbalancing specialization by promoting the integration of scientific knowledge.” Insisting, as Dupré, Cartwright, and Teller do, that science cannot be unified, helps combat reductionist ideals, but does little to shed light on why integration is still seen as a worthy goal.

How can we understand the unifying tendency in science without returning to reductionism? One option is to defend non-reductive unity at a metaphysical level: examples of this are non-reductive physicalism and the idea of the “primacy of physics” defended by Ladyman and Ross (8). Such metaphysical discussions will be avoided in the current paper, in order to focus on the methodology and the products of science. Among methodologically inclined philosopher of science, there seems to be an emerging tendency to revive a Neurathian use of the term “unity of science,” as some of the authors in Symons et al. (9) do. But more influential, especially among scientists themselves, has been the adoption of a new term of art for what is at bottom the same idea of unifying different research approaches: explanatory pluralism. It is this term and the debate surrounding it that we will focus on.

The term “explanatory pluralism” is not without problems, one of which is that different authors use it in sometimes quite different ways. But we can glean the core idea from some representative citations, all of them from papers which set out to defend a form of explanatory pluralism within the psychological sciences:

Explanatory pluralism holds that simultaneously pursuing research at multiple analytical levels in science tends to aid progress at each of those levels [(10), p. 738]

Explanatory pluralism hypothesizes multiple mutually informative perspectives with which to approach natural phenomena [(11), p. 436]

On this view, different sciences have a degree of autonomy (they are not to be eliminated), but also interact in an effort to understand physical reality at different scales (they are not fully autonomous silos). […] different sciences and theoretical approaches should maintain their emphasis on different proprietary scales but should also work to unify their work as much as possible, insofar as they often describe the same phenomena in different but compatible ways [(12), p. 3]

As we can see, there are two elements to the core idea: first, that science can be broken up into distinct enterprises, and, second, that it is scientifically fruitful to have interaction between these enterprises. The authors have different ideas about how to carve up science: in terms of “levels,” or “perspectives,” or “scales,” or simply “sciences”; but in each case, we are presumably to identify them with well-known disciplines and subdisciplines, such as high-energy physics, cell biology, neuroscience, and cognitive psychology. We will ignore the question of how exactly to carve up science and assume that speaking of research approaches is clear enough.

More relevant to our current purposes is the second element of explanatory pluralism, namely, the idea that the sciences have to interact in order to achieve their full potential. As we can see, this claim is formulated in different ways by the different authors. McCauley and Bechtel frame it as a prediction about the rate of scientific progress, although one that is not, perhaps, especially clear, since it is not evident which contrast they are drawing. Kendler formulates explanatory pluralism as a methodological norm and contrasts it with reductionism. Abney et al. take explanatory pluralism to be an alternative not only to reductionism but also to an isolationist view of science that they attribute – perhaps inaccurately, since his article opposes reduction but not interaction in general – to Fodor (4). Given our interest in finding a middle ground between the unity and the disunity of science, this is an especially interesting version of the explanatory pluralism. But the formulation of Abney et al. remains vague: it exhorts us to unify the sciences “as much as possible,” but it does not indicate how far that possibility extends.

Some of the most thoughtful analyses of explanatory pluralism are those of Marchionni (13), Mitchell (14), Campaner (15), and Van Bouwel (16). [Closely related, though couched in a different terminology and less focused on the technical details of explanation, is Brigandt (17) account of *explanatory integration* as an intermediate between reductionism and pluralism.] All these authors take the view that explanatory pluralism is primarily about what *explanations* the best science will end up with, and more precisely about the question whether explanations from different research approaches can all be integrated into a coherent whole.

Marchionni (13) makes a distinction between two ways in which explanations of the same phenomenon on a macro and micro level can complement each other: *weak complementarity*, which holds when the two explanations are both legitimate and autonomous, but cannot be combined; and *strong complementarity*, which holds when the two explanations can be integrated into a whole that provides a better explanation than the two explanations did separately. If weak complementarity holds, we have two research approaches that are essentially independent; this is a disunified or isolationist view of science. When strong complementarity holds, our best understanding of the world is generated when two or more research approaches interact: this is a unified or integrationist view. We thus arrive at a gliding scale ranging from the ultimate unity that is reduction/­monism, to the ultimate disunity that is weak complementarity/isolationism, with strong complementarity/integrationism in between.

However, the idea of strong complementarity involves a certain instability. On the one hand, it poses different, distinct research approaches; and on the other hand, it tells us that the results of these approaches can be put together to form a single picture of the world, a picture that is more enlightening than any of the separate pictures. But if the sciences are to be integrated so tightly and do not have an autonomous domain of knowledge wherein they reign supreme, in what sense can they still be said to be distinct? Do they not reveal themselves as merely different parts of the same one-and-only scientific discipline?

This seems to be the background against which Campaner (15), in an attempt to explain how different kinds of psychiatric explanation can be combined, asks the following pertinent questions about explanatory pluralism:

Is there any underlying idea that some sort of complete explanatory picture can be – sooner or later – ­elaborated, or is some more radical form of pluralism advanced here? Is pluralism suggested here as only the acknowledgement of the existence and toleration of a diversity of current explanatory theories, or also as the idea that distinctive views will persist as such in the long run? In other terms, is actual plurality treated in this context as provisional and resolvable, or is the idea that renouncing pluralism would lead to some loss of explanatory information? (pp. 98–99)

Unlike Marchionni, who comes out in favor of strong complementarity, Campaner believes that the different types of explanation in psychiatry will turn out to be impossible to integrate into a single type of explanation. She points at the very different aims and interests of different actors in the field of psychiatry, and she argues that there is little reason to suppose that the explanations constructed to advance those different interests will coincide, even in the long run. According to her, we must be “open to the possibility that, at least in principle, explanatory pluralism can be a permanent state” (idem, p. 101), where explanatory pluralism is here understood – justifiably, but somewhat confusingly when compared to Abney et al. – as the isolationist rather than the integrationist position.

Van Bouwel (16), in a commentary on Campaner and using and expanding the earlier classification of Mitchell (14), adds another level of sophistication to the analysis. Next to explanatory reductionism, Van Bouwel distinguishes no fewer than five different kinds of explanatory pluralism, ranging from the more monistic to the more pluralistic:

1. *Explanatory reductionism*: there is a single privileged research approaches, and ultimately the best understanding of the world will be achieved when all the explanations from other approaches are reduced to this privileged approach.
2. *Temporary pluralism*: it is methodologically advisable to promote a temporary plurality of competing theories, as a means of achieving, in the end, one single unified theory that gives the best explanations.
3. *Integrative pluralism*: satisfactory explanations can only be generated by integrating the findings of different research approaches. (This is equivalent to always embracing Marchionni’s idea of strong complementarity.)
4. *Interactive pluralism*: research approaches often generate satisfactory explanations by themselves, but it is also often – though not invariably – the case that the integration of explanations from different sciences leads to a better explanation. (This position, which is Van Bouwel’s preferred position, posits a mixture of Marchionni’s two kinds of complementarity.)
5. *Isolationist pluralism*: different research approaches generate very different kinds of explanation, which are all valid but cannot be integrated. (This is equivalent to always embracing Marchionni’s idea of weak complementarity.)
6. *Anything goes pluralism*: all theories and perspectives are equally valid, and the greatest understanding of the world is achieved by an unlimited proliferation of theories and perspectives.

Van Bouwel is undoubtedly right when he suggests that it would be tough to defend either the idea that isolation is always correct or the idea that integration is always correct. Interactive pluralism, which decides on a case-to-case basis whether integration will succeed or whether isolation is needed, seems to be the most rational position. But in its relaxed wait-and-see attitude, it misses out on something that is more adequately captured by the admonitions of McCauley, Bechtel, Kendler, and Abney et al. all of whom push toward integration. There is a methodological presumption in science in favor of integration: where we *can* integrate, one feels, we *should* integrate; after all, pushing toward integration has led to many great advances.^1^ The scientist who insists on the splendid isolation of her discipline will come under immediate suspicion for being, perhaps, too conservative. “Interdisciplinarity” remains a word with which one can woo funding agencies. In other words, we love the unity of science, we are striving toward the unity of science, and if we fail to achieve it – that is, more specifically, if we fail to achieve an integrative pluralism where all the sciences work together to create one single coherent explanation of every phenomenon – than there must be some particular obstacle in the way of that integration. It is that obstacle that I wish to consider. Is the scientist who believes that her explanations stand alone and cannot fruitfully be combined with those of other sciences automatically an unintelligent conservative, or are there circumstances under which it is rational to embrace an isolationist pluralism? What, we may ask more specifically, are the circumstances under which two explanations can fail to be combinable into a single, more complete explanation?

Answering that question will be the burden of the Section “Combining Explanations” of this paper. But before I embark on that project, it will be useful to mention Van Bouwel’s own approach to this question and distinguish my project from his. According to Van Bouwel et al. (19):

[e]xplanatory pluralism consists in the claims that (i) the best form (and level) of explanation depends on the kind of question one is willing to answer by the explanation and (ii) that in order to answer all explanation-seeking questions in the best way possible we will need more than one form (and level) of explanation (p. 36)

The approach championed by these authors, which also influences Gervais’ (20) account of inter-level explanations, starts not from a phenomenon, to then ask whether different research approaches should cooperate in giving a single explanation of that phenomenon, but starts from the idea that different epistemic interests lead to different explanatory questions that are best answered by explanations involving different forms and levels. This more pragmatic approach to explanation leads to a natural answer to the questions I posed above: yes, one can say, it is rational to believe that isolation is sometimes the best strategy, because under some circumstances narrow isolated explanations are more conducive to our specific epistemic goals than grand integrative stories. [In their 2011 article, Van Bouwel et al. (19) are actually concerned with showing that reductive explanations have a place in science next to high-level explanations, but I take it that they would also agree with the approach to isolation I just outlined.] Actual examples of science can then be used to prove that scientists indeed choose between integration and isolation based on pragmatic and contextual factors.

I have no quarrel with such an approach. Suppose, for a moment, that there is indeed a single best, completely integrated explanation of any phenomenon. Then, it is undoubtedly true – and I would expect even hard reductionists to agree – that there are strong pragmatic reasons against using this explanation to answer any and all questions about that phenomenon. A therapist interested in curing her patient’s depression might not need to hear about the details of the patient’s neurochemistry in order to prescribe the right cure, while the patient’s company doctor might need to know nothing at all about the causes of the depression in order to decide whether or not to grant the patient extended sick leave. In practical contexts, the “best” explanation is often simple and idealized. And it would also be true, as the pragmatist might stress, that in practice we tend to lose important insights and information if we do not keep our practical goals in mind from the start, so that there is a more fundamental, if still practical, reason for pursuing isolated rather than integrated explanations.

So, even if it were true that there is a single best, completely integrated explanation of any phenomenon – where “best” is understood not in a pragmatic and contextual way, but in terms of an ideal state of understanding – there are still legitimate practical concerns about integration. But I want to know whether that supposition, which seems to underlie much of the theoretical defense of integration, is true. If it is, then the sciences are fundamentally one, at least as far as explanation is concerned; and we will reach the most perfect understanding of the world when we relentlessly pursue integration. If not, then the sciences are fundamentally a plurality; and we will lose some understanding if we push our quest for integration too far.

## Combining Explanations

Is there a single best, completely integrated explanation of any phenomenon? There are instances where one might doubt this for reasons having to do with what the explanations are *about*. For instance, one might doubt whether explanations involving the mind and explanations involving the body could ever be combined; or explanations involving facts and explanations involving values. These doubts are related to some of the thorniest metaphysical issues in all of philosophy. We will sidestep these issues – which we could not possibly do justice to here – and focus instead, not on what explanations are *about*, but on the general *form* or structure of explanations. What I want to know is what general feature of two explanations of the same phenomenon could stand in the way of their being combined into a single bigger explanation.

In order to simplify the discussion, I will make two assumptions. First, I will assume that the things that get explained by explanations – with a technical term, the *explananda* – are facts, and that these facts can be put into a contrastive form, that is, an “A rather than B” form. An explanation thus may explain why Tom is depressed rather than not being depressed; or why he is depressed rather than manic; or why he has been depressed since August rather than having been depressed for a longer or shorter time. Not much in the discussion will hinge on this assumption, but settling on one specific form of explanandum will increase both brevity and clarity. In addition, it has been made by many authors working on scientific explanation, from Van Fraassen (21) to Woodward (22).

When we start thinking about features of explanations that could stand in the way of their being combined, one rather trivial feature will come to us immediately: logical inconsistency. If I explain Tom’s depression from that fact that he has been working too much and you explain it from the fact that he has been jobless, we are contradicting each other and no integration is possible. In order to avoid this, I will stipulate that in all the examples to be discussed later on, the explanations given are true; and furthermore, I assume – this is my second substantive assumption – that true statements are always logically compatible. Many will regard this assumption as a self-evident truth; I myself do not; but I will assume its truth here in order to focus on the issues at hand.

Given this second assumption, there seems to be a strong presumption in favor of the idea that all explanations of the same explanandum will be combinable. After all, we can simply put them together; there being no logical incompatibility, nothing could stop us from doing so. This, I take it, is precisely why integrative approaches to science are so intuitively persuasive: if all our final theories are true, it surely *must* be possible to combine them. But of course, there are many ways to “combine” explanations, and it behooves us to take stock of them – and of any presuppositions they entail – before coming to a judgment about the matter.

In the following, I will identify three ways in which explanations can be combined: by presenting additive causes, by presenting different parts of a single causal tree, or by describing supervening levels. After a brief discussion of these three kinds of compatibility, I will argue that all of them share a basic presupposition that I will call *counterfactual compatibility*. This will suggest a way that even true, logically consistent explanations of the same fact can fail to be combinable: by *counterfactual incompatibility*.

As our example explanandum, let us take the fact F that patient P suffers from major depressive disorder (MDD), rather than not suffering from it. Let us postulate that P’s MDD can be causally linked to a life history that has led to self-esteem and relationship issues; that the depression has been triggered by the loss of a job and the death of his best friend; that on a neural level the depressive symptoms are caused by, among other things, a disruption of neuroplasticity; and that P’s self-esteem issues can be related to the exaggerated expectations his authoritarian father had of his only son. Given this situation, both of the following are acceptable explanations of F:

(1) P suffers from MDD because he lost his job.
(2) P suffers from MDD because his best friend died. These explanations both present causal factors that increased the likelihood of a depression and were in fact causally linked to it. Irrespective of whether either of them was sufficient for the occurrence of MDD, or whether both together were needed to trigger it, these causes can be added to each other in a single, more encompassing explanation:
(3) P suffers from MDD because he lost his job and his best friend died. This is what I call the *presentation of additive causes*: when two or more explanations present different causal factors that are independent but both increase the probability of the explanandum, we can simply combine them into a single conjunctive causal factor that is more informative than either of the factors alone. Of course, it is also possible that a set of explanations presents causal factors that are not independent, but that depend on each other because they are causally linked. Take, for instance, the following:
(4) P suffers from MDD because he lost his job and has been unable to find a new one.
(5) P suffers from MDD because he has self-esteem issues, which made him ineffective in his last job and caused him to lose it. The loss of his job triggered MDD.
(6) P suffers from MDD because the economy is in a slump and that has made him unable to find a new job. If he had found a new job soon after losing his last one, MDD would not have been triggered.

The relationship between these explanations is that each of them traces out a different part of a single causal *tree*, where a causal tree is the structure that is generated by providing the direct causes of one event, and then continuing to provide causes for any event in the tree whose causes have not been given yet. In this case, (4) explains F by giving two of its causes: the loss of the job and the inability to find a new one. (5) explains F by giving only one of those causes – the loss of the job – but by also explaining what caused that cause, thus moving up a level in the explanatory tree. Explanation (6) gives another of the causes of F – the inability to find a new job – and gives the causes of that cause. It is of course possible to combine (4–6) into a single, more complete description of the explanatory tree:

(7) P suffers from MDD because he has self-esteem issues and because the economy is in a slump. The self-esteem issues caused him to be ineffective at his last job, which in turn caused him to be fired. Because of the economic slump, he has been unable to find a new job. The prolonged joblessness was one of the things that triggered P’s current episode of MDD.

This is what I call the *presentation of different parts of a causal tree*. Of course, the addition of causes and the presentation of different parts of a causal tree can be combined more or less *ad infinitum* in order to trace out the entire causal history of the event in the explanandum. Each of the explanations gives a different part of the tree, gives us a different set of events and causal links between them, and as this proceeds, we know about a larger part of the tree and understand the explanandum better.

These two ways of combining explanations are straightforward and important in practice, but they pose few theoretical problems. Things become more interesting when we move to two explanations like these:

(8) P suffers from MDD because he has a high stress level and stress causes the symptoms known as depression.
(9) P suffers from MDD because he has abnormal levels of cortisol, serotonin, and norepinephrine. These abnormal levels reduce the neuroplasticity of P’s brain, which in turn causes the symptoms known as depression.^2^

We cannot understand (8) and (9) as tracing out different parts of a causal tree, for the simple reason that – at least on standard theories of the mental – they trace out the *same* part, but described at different levels or in different vocabularies. Where (8) speaks about stress, (9) speaks about the abnormal levels of certain hormones, but these are two descriptions of the same state. It is both possible and enlightening to combine (8) and (9):

(10) P suffers from MDD because he has a high stress level, which involves him having abnormal levels of cortisol, serotonin, and norepinephrine. These abnormal levels reduce the neuroplasticity of P’s brain, which in turn causes the symptoms known as depression.

Philosophical questions about this situation remain, especially about the status of the word “involves” in (10). Is having stress *identical* to having certain hormonal levels, or does having stress instead *supervene*^3^ on hormone levels? If it supervenes, could there be a reduction of theories about stress to theories about hormones, or are reductions impossible? Might it even be the case that this description of the situation is wrong, and that stress and certain hormone levels are merely accidentally cooccurring? Such questions are familiar from the philosophy of mind and will not be resolved any time soon. But for our current discussion, it turns out, perhaps surprisingly, that the answers to these questions make no difference. On any of the options in the debate, either (8) and (9) can be combined into (10) or at least one of them is false:

• On a reductionist theory, the two explanations are simply saying the same things in different vocabularies; once this is seen, the combination is trivial, because it turns out that there is nothing to combine.
• On a non-reductionist theory which sees psychological notions like “stress” as supervening on neurochemical states, the two explanations can both be given, and then linked through supervenience relations to result in a more complete explanation. This is what I will call the *description of supervening levels*.
• On a non-reductionist theory that rejects the supervenience thesis and instead believes that psychological events such as stress and neurochemical events such as high hormone levels are wholly distinct but related through the relation of causation, the two explanations can be combined by giving the causal interrelations between them. In this case, combining (8) and (9) into (10) turns out to be a case of *presentation of different parts of a causal tree*.
• On a radical dualist theory which sees mental events like stress and physical events like hormone levels as wholly distinct and non-interacting, explanation (9) must be false, for hormone levels cannot cause stress. So in this case, too, we do not have two true explanations of the same phenomenon that cannot be combined; we have a true and a false explanation, and the false explanation must be rejected.

Which of these options is correct will be highly relevant to our view of the relation between psychology and neuroscience. But what anyone can seemingly agree on is that once we have found the true explanations, those explanations *can* be combined into a single story – either by identifying them, by linking them through supervenience relation, or by linking them through causal relations.

Having seen three important ways in which true explanations can be combined, and are combined in practice, we are still faced with the question of whether there are any conditions under which they cannot. To answer that question, we must think about what explanations are and how something could fail to be an explanation, even though its parts are explanations.

When we do think through the properties of explanations, we quickly find that they are not merely lists of unconnected facts. Explanations always trace links between the fact to be explained and other facts. Different theories of explanation have different ideas about what these links are like: according to Hempel’s original DN-model, explanations show how the fact to be explained can be derived from other facts through laws of nature; according to unificationist theories, explanations show how the fact to be explained can be derived using unifying arguments; according to causal theories of explanation, explanations explain a fact by giving its causal antecedents [see Salmon (25) and Woodward (26) for overviews]. But what all these theories have in common, and what is indeed one of the central facts about explanation that any theory of explanation would have to do justice to, is that explanations allow us to draw *counterfactual conclusions* about the explanandum. To *know that* P suffers from MDD is to know something important; but to *understand why* P suffers from MDD is to have, in addition, a measure of insight into the conditions under which he would *not* have suffered. Explanations allow us to make claims about what *would have happened* in different circumstances. And this is indeed one of the prime reasons that we are interested in explanations at all, for by allowing us to see what would happen in different circumstances, they allows us to make an informed choice between different courses of action. [For more on the relation between explanation, causation, and counterfactuals, see Chapter 3 of Woodward (22).]

If one of the central obligations on an explanation is to allow us to draw counterfactual conclusions about the explanandum, then it is reasonable for us to require explanations to fulfill that obligation. To be precise, it is reasonable to ask of any explanation that the counterfactual consequences that follow from it are consistent: that is, that we cannot show from it both that if A had happened, C would have happened; and that if A had happened, C would not have happened. In other words, the counterfactual picture painted by any explanation should be coherent.

This in turn suggests a condition that two explanations of the same fact have to fulfill in order to be combinable into a single explanation: they should not have logically incompatible counterfactual consequences. If they do not, we will call them *counterfactually compatible*. If, on the other hand, they do have logically incompatible counterfactual consequences, we will call them *counterfactually incompatible*. The claim I am making, then, is that two true explanations of the same fact are combinable into one explanation only if they are counterfactually compatible. (This is a necessary condition. Perhaps it is also sufficient, but I have no argument to that effect.)

In all our previous examples, the explanations were indeed counterfactually compatible. Both (4) and (5) imply that if P had not lost his job, he would not have had MDD. In addition, (5) implies that if P had not had self-esteem issues, he would not have lost his job; this is of course compatible with the previous claim. Both (4) and (6) imply that if P had been able to find a new job soon, he would not have had MDD. In the case of (8) and (9), the counterfactual implications are different but logically compatible: (8) implies that P would not have suffered from MDD if he had not suffered from stress, whereas (9) implies that P would not have suffered from MDD if his hormone levels had been normal; and these two claims are perfectly consistent on both reductive and non-reductive theories of the mental.

We must now ask ourselves whether it is ever possible for two true explanations to be counterfactually incompatible. Let us first look at an example involving two very different explanations of the same fact, one from the perspective of textbook physics and one from the perspective of common sense teleology. Suppose that a door in my living room is open rather than closed. Why? Here are two explanations:

(11) The door is open because a force greater than F was applied to it from the inside while the handle was down.
(12) The door is open to allow fresh air to get in.

Both of these explanations can be true at the same time. But now let us ask the following question: would this door have been open if it had been a door to the cellar instead of a door to the garden? The physicist, with (11) in hand, would say that, yes, the door would still have been open. After all, cellar doors do not have physical properties that make them physically more difficult to open than garden doors. But the common sense thinker, looking at (12), would say no, the door would have been closed if it had been a cellar door. After all, cellar doors are not opened to let in fresh air. Who of the two is right? Would this door have been open if it had been a door to the cellar? Well, yes *and* no – it depends on the perspective we are taking. But this means that the explanations from the two perspectives, while both valid and true, fail the test of counterfactual compatibility and cannot be combined into a single coherent explanation.

One might object that any incompatibility here is the result of the incompatibility of a broadly causal and a broadly teleological perspective; and one might then go on to claim that teleology has no place in science. If that is true, then examples like the one above could show at most that science cannot always be integrated with common sense; but this does not disprove the integrationist claim that the sciences themselves are always capable of being integrated. Perhaps this is true; although it would already be an interesting result, since discussions about teleology are by no means dead in science. But counterfactual incompatibility can in fact also arise between two perspectives that are both purely causal.

Let us return to our poor patient P, and let us ask the following question: suppose that P had been a woman, would he still have suffered from MDD? One way to approach this question – the approach that would be favored by a neuroscientist – would be to review the differences between male and female brains. Let us supposes that there is no systematic difference between the sexes such that female brains handle abnormal levels of cortisol, serotonin, and norepinephrine differently from male brains. Then, the neuroscientist would pronounce, correctly and with ample justification, that if P had been female, (s)he would still have suffered from MDD.

But the question could also be answered by P’s therapist, who has been especially interested in talking through his life history with him, with a special emphasis on traumatic events from his early childhood. According to this therapist the crucial cause of P’s self-esteem issues is the way P’s father treated his only son; a way that was markedly different from the way he treated his daughters. The therapist thus comes to the conclusion – just as correct and just as justified as that reached by neuroscientist – that if P had been a woman, (s)he would not have suffered from MDD.

There is nothing especially mysterious about this situation. Different scientific perspectives on P naturally lead to different ways of evaluating counterfactual claims about him. For a neuroscientist, contemplating the influence of gender means contemplating the way that gender has influenced the structure and functioning of the patient’s brain. For the therapist, contemplating the influence of gender means contemplating the way that gender has influenced the patient’s life history. Both of these perspectives are equally valid, and both lead to explanations that should be accepted. But these explanations cannot be accepted into one single coherent explanation; for combining them leads to a story in which P would both have suffered from MDD and not suffered from MDD if he had been a woman. So, the therapist’s life-history approach and the neuroscientist’s approach have to remain isolated to a certain extent. Here, we have a case of counterfactual incompatibility; and in general, counterfactual incompatibility may occur when we try to integrate explanations from different research approaches. When it does, it acts as a barrier to integrative pluralism.

This conclusion could be attacked in two ways. First, one could attack the claim that counterfactual compatibility is a requirement for two explanations to be combined. Now, admittedly, by choosing a suitable low standard for what “integration” means, one can always claim that two research approaches can be integrated. But counterfactual incompatibility is a real barrier to any substantive kind of integration, because it means that we cannot simply transfer conclusion reached in one approach to the other approach. If the neuroscientist finds that gender is irrelevant to MDD, the therapist or the sociologist cannot just accept that conclusion; for the conclusion, while true – in our example – from the neuroscientific perspective, might well be false from the other perspectives. This non-transferability of counterfactual conclusions is surely a good reason to hold that the different research approaches are to some extent isolated and autonomous.

Second, one could claim that, my examples notwithstanding, counterfactual compatibility cannot occur between true explanations. For, one could argue, it is logically impossible that “if A had happened, then B would have happened” and “if A had happened, then B would not have happened” are both true. To substantiate this conclusion, one could appeal to influential theories about the truth conditions of counterfactuals. Lewis (27), for instance, tells us that “if A had happened, then B would have happened” is true just in case that B is true in the closest possible world where A is true, where the closeness of possible worlds is defined in terms of their similarity to ours. If such a story were correct, and, crucially, *if similarity were a non-contextual affair*, something that should be evaluated in the same way across all the sciences, then either the therapist or the neuroscientist would have to be wrong. To see which, we would have to find out which world is more similar to ours, the one envisaged by the therapist or the one envisaged by the neuroscientist. And whichever of them in their imaginative flights stayed closer to home, so to speak, would be the person drawing the correct counterfactual conclusions.

Such a procedure, however, has very little to recommend itself. Theories about the truth conditions of counterfactuals should respect our everyday evaluations of counterfactuals; and it is an undeniable fact that people working from different perspectives use different scenarios to evaluate the same counterfactual claims. As Lowe (28) points out, the truth conditions of counterfactuals are highly context-dependent. Lowe then argues (pp. 54–55) that the context influences how we evaluate claims about the similarity of possible worlds, and that this context is at least partly defined by the intentions of the speaker. For our current purposes, we can slightly modify his proposal and state that the context within which counterfactuals are evaluated is at least partly defined by the research approach within which the claim appears. Counterfactual evaluation in neuroscience takes scenarios into account that are ignored in the therapeutic setting, and the other way around. The different sciences use different relevance criteria; and this does not make a difference not only for which facts they uncover but also for how they reason about counterfactual scenarios. Since explanations are tightly connected to counterfactual scenarios, these differences between research approaches translate into an incompatibility of the explanations they generate.

This concludes my argument for the claim that I set out to prove, namely, that true explanations of the same fact sometimes cannot be combined into a single bigger explanations. Counterfactual incompatibility is a barrier to such combination, and counterfactual incompatibility is real. This result nicely mirrors that of Lange (29). His point is that different research approaches take different sets of counterfactuals seriously, and that this leads to incompatible laws; my point is that different research approaches sometimes reach incompatible results when evaluating identical counterfactuals. Both points support the conclusion that research approaches can be expected to be at least partly autonomous.

Let me end this section by professing ignorance about two points. First, I am not sure whether counterfactual incompatibility can also occur within a single research approach – e.g., whether two true neurological explanations of a brain phenomenon could ever turn out to be incompatible. If this were possible, science would be even more disunified than we tend to think. Second, I do not know whether this section has covered all the ways in which explanations can be combinable or fail to be combinable. In this respect, I make no claim to having exhausted the territory.

## Conclusion

My analysis of the debate surrounding the unity of science and explanatory pluralism revealed that the most pressing issue lies in the choice between integrative and isolationist pluralism; or rather, in finding out whether the integrative tendencies present in current science should be fully indulged in, or should be held in check by affirming that a certain amount of isolation is unavoidable. I further distilled this issue into the question of whether two true explanations of the same fact could ever fail to be combinable into one single explanation. It turns out that although many explanations are in fact combinable, this only holds when they have compatible counterfactual consequences. I then argued that true explanations from different sciences can have incompatible counterfactual consequences. This leads us to the general conclusion that a certain amount of isolation between the sciences is indeed both present and unavoidable; forcing all the sciences to use the counterfactual relevance criteria of one of them would rob us of part of the insight that the different sciences can give us and would lead to the uncritical transfer of counterfactual claims from one science into another, with potentially disastrous results (in the case of, e.g., a sociologist who rejects the possibility that gender could be related to psychological conditions because the neuroscientists tell him that there is no such relation). This does not mean that we should not strive for integration and the benefits of interdisciplinarity. But it does mean that we should not take this project too far, for, at the end of the day, there will still be the different sciences with their different perspectives and insights. The plurality of the sciences is to be cherished rather than combated.

## Author Contributions

The author confirms being the sole contributor of this work and approved it for publication.

## Conflict of Interest Statement

The author declares that the research was conducted in the absence of any commercial or financial relationships that could be construed as a potential conflict of interest.

## Footnotes

^1^See also Andler (18), pp. 140–141, for an appraisal of why we cannot ignore the unifying tendencies in science. Of course, there are critics of integration and interdisciplinarity too, but I venture – although I have no hard data to back this up – that most of these critics have doubts about the *possibility* of integration, rather than about the *desirability* of integration where this is possible.

^2^For the potential relation between stress hormones, neuroplasticity, and depression, see Maletic et al. (23) and Pittenger and Duman (24).

^3^Supervenience is a notoriously difficult term to define adequately, but in this article, I will take it to be the relation such that (a) the values of supervening properties at time *t* are fully determined by the values at time *t* of the properties they supervene on, but (b) the supervening properties cannot be identified with the supervened-on properties. Many philosophers have defended the idea that while, say, mental states are not identical to brain states; nevertheless, our brain states fully determine our mental states. If this is so, then mental states supervene on brain states in the sense I am using the term here.

## References

[1] Cat J The unity of science. Winter 2014 ed In: Zalta EN, editor. The Stanford Encyclopedia of Philosophy (2014). Available from: http://plato.stanford.edu/archives/win2014/entries/scientific-unity/

[2] Pombo O Symons J Torres JM Neurath and the unity of science: an introduction. In: Symons J Pombo O Torres JM, editors. Otto Neurath and the Unity of Science. Dordrecht: Springer (2011). p. 1–11.

[3] Oppenheim P Putnam H The unity of science as a working hypothesis. In: Feigl H Scriven M Maxwell G, editors. Minnesota Studies in the Philosophy of Science (Vol. 2), Minneapolis: Minnesota University Press (1958).

[4] Fodor J Special sciences: or the disunity of science as a working hypothesis. Synthese (1974) 28:97–115. 10.1007/BF00485230

[5] Dupré J The Disorder of Things. Metaphysical Foundations of the Disunity of Science. Cambridge, MA: Harvard University Press (1993).

[6] Cartwright N The Dappled World. A Study of the Boundaries of Science. Cambridge: Cambridge University Press (1999).

[7] Teller P Twilight of the perfect model model. Erkenntnis (2001) 55:393–415. 10.1023/A:1013349314515

[8] Ladyman J Ross D Every Thing Must Go. Metaphysics Naturalized. Oxford: Oxford University Press (2007).

[9] Symons J Pombo O Torres JM, editors. Otto Neurath and the Unity of Science. Dordrecht: Springer (2011).

[10] McCauley RN Bechtel W Explanatory pluralism and heuristic identity theory. Theory Psychol (2001) 11:736–60. 10.1177/095935430­1116002

[11] Kendler KS. Toward a philosophical structure for psychiatry. Am J Psychiatry (2005) 162:433–40. 10.1176/appi.ajp.162.3.433 15741457

[12] Abney DH Dale R Yoshimi J Kello CT Tylén K Fusaroli R. Joint perceptual decision-making: a case study in explanatory pluralism. Front Psychol (2014) 5:330. 10.3389/fpsyg.2014.00330 24795679 PMC4006048

[13] Marchionni C Explanatory pluralism and complementarity: from autonomy to integration. Philos Soc Sci (2008) 38:314–33. 10.1177/0048393108319399

[14] Mitchell S Unsimple Truths. Science, Complexity, and Policy. Chicago: University of Chicago Press (2009).

[15] Campaner R Explanatory pluralism in psychiatry: what are we pluralists about, and why? In: Galavotti MC, editors. New Directions in the Philosophy of Science. Cham: Springer (2014). p. 87–103.

[16] Van Bouwel J Pluralists about pluralism? Different versions of explanatory pluralism in psychiatry. In: Galavotti MC, editors. New Directions in the Philosophy of Science. Cham: Springer (2014). p. 105–19.

[17] Brigandt I Beyond reduction and pluralism: toward an epistemology of explanatory integration in biology. Erkenntnis. (2010) 73:295–311. 10.1007/s10670-010-9233-3

[18] Andler D Unity without myths. In: Symons J Pombo O Torres JM, editors. Otto Neurath and the Unity of Science. Dordrecht: Springer (2011). p. 129–44.

[19] Van Bouwel J Weber E De Vreese L Indispensability arguments in favour of reductive explanations. J Gen Philos Sci (2011) 42:33–46. 10.1007/s10838-011-9141-5

[20] Gervais R. A framework for inter-level explanations: outlines for a new explanatory pluralism. Stud Hist Philos Sci (2014) 48:1–9. 10.1016/j.shpsa.2014.07.002 25571740

[21] Van Fraassen B The Scientific Image. Oxford: Oxford University Press (1980).

[22] Woodward J Making Things Happen. Oxford: Oxford University Press (2003).

[23] Maletic V Robinson M Oakes T Iyengar S Ball SG Russell J. Neurobiology of depression: an integrated view of key findings. Int J Clin Pract (2007) 61:2030–40. 10.1111/j.1742-1241.2007.01602.x 17944926 PMC2228409

[24] Pittenger C Duman RS. Stress, depression, and neuroplasticity: a convergence of mechanisms. Neuropsychopharmacology (2008) 33:88–109. 10.1038/sj.npp.1301574 17851537

[25] Salmon W Four Decades of Scientific Explanation. Minneapolis: University of Minnesota Press (1989).

[26] Woodward J Scientific explanation. Winter 2014 ed In: Zalta EN, editor. The Stanford Encyclopedia of Philosophy (2014). Available from: http://plato.stanford.edu/archives/win2014/entries/scientific-explanation/

[27] Lewis D Counterfactuals. Oxford; Cambridge, MA: Blackwell Publishers; Harvard University Press (1973).

[28] Lowe EJ The truth about counterfactuals. Philos Q (1995) 45:41–59. 10.2307/2219847

[29] Lange M Who’s afraid of *Ceteris-Paribus* laws? Or: how I learned to stop worrying and love them. Erkenntnis (2002) 57:407–23. 10.1023/A:1021546731582

